# Profiling expression of coding genes, long noncoding RNA, and circular RNA in lung adenocarcinoma by ribosomal RNA‐depleted RNA sequencing

**DOI:** 10.1002/2211-5463.12397

**Published:** 2018-02-21

**Authors:** Xiangxiang Ding, Shuai Zhang, Xiao Li, Changjiang Feng, Qi Huang, Shaodong Wang, Siwei Wang, Wenjia Xia, Fan Yang, Rong Yin, Lin Xu, Mantang Qiu, Ming Li, Jun Wang

**Affiliations:** ^1^ Department of Radiology Jiangsu Cancer Hospital Jiangsu Institute of Cancer Research Nanjing Medical University Affiliated Cancer Hospital Nanjing China; ^2^ Department of Thoracic Surgery Jiangsu Key Laboratory of Molecular and Translational Cancer Research Jiangsu Cancer Hospital, Jiangsu Institute of Cancer Research Nanjing Medical University Affiliated Cancer Hospital Nanjing China; ^3^ Department of Thoracic Surgery Peking University People's Hospital Beijing China; ^4^ Department of Thoracic Surgery The Chinese People's Liberation Army General Hospital Beijing China

**Keywords:** circular RNA, long noncoding RNA, lung adenocarcinoma, RNA sequencing

## Abstract

Noncoding RNA play important roles in various biological processes and diseases, including cancer. The expression profile of circular RNA (circRNA) has not been systematically investigated in lung adenocarcinoma (LUAD). In this study, we performed genomewide transcriptome profiling of coding genes, long noncoding RNA (lncRNA), and circRNA in paired LUAD and nontumor tissues by ribosomal RNA‐depleted RNA sequencing. The detected reads were first mapped to the human genome to analyze expression of coding genes and lncRNA, while the unmapped reads were subjected to a circRNA prediction algorithm to identify circRNA candidates. We identified 1282 differentially expressed coding genes in LUAD. Expression of 19 023 lncRNA was detected, of which 244 lncRNAs were differentially expressed in LUAD. AFAP1‐AS1, BLACAT1, LOC101928245, and FENDRR were most differentially expressed lncRNAs in LUAD. Also identified were 9340 circRNA candidates with ≥ 2 backspliced, including 3590 novel circRNA transcripts. The median length of circRNA was ~ 530 nt. CircRNA are often of low abundance, and more than half of circRNAs we identified had < 10 reads. Agarose electrophoresis and Sanger sequencing were used to confirm that four candidate circRNA were truly circular. Our results characterized the expression profile of coding genes, lncRNA, and circRNA in LUAD; 9340 circRNAs were detected, demonstrating that circRNA are widely expressed in LUAD.

**Database:**

The raw RNA sequencing data have been submitted to Gene Expression Omnibus (GEO) database and can be accessed with the ID GEO: http://www.ncbi.nlm.nih.gov/geo/query/acc.cgi?acc=GSE104854.

**Abbreviations**
circRNA: circular RNA
FC: fold change
FDR: false discovery rate
GO: gene ontology
KEGG: Kyoto encyclopedia of genes and genomes
lncRNA: long noncoding RNA
LUAD: lung adenocarcinoma
rRNA: ribosome RNA

The ENCODE project reveals human genome is actively transcribed; however, coding genes only account for only 1.5%–2% of human transcripts and the vast majority are noncoding RNA 1, 2. Increasing evidence has proved that noncoding RNAs are involved in almost every aspect of biological or pathological processes 3, 4, 5. Various kinds of functional noncoding RNAs have been identified, like snRNA piwiRNA, microRNA, long noncoding RNA (lncRNA), and circular RNA (circRNA) 6, 7, 8, 9. Among them, lncRNA and circRNA have been the focal points of research, although they were discovered decades ago. LncRNAs are a kind of RNA transcripts larger than 200 nt and without coding capacity. A lot of cancer‐associated lncRNAs have been characterized, such as HOTAIR, CCAT2, PVT1, and ANRIL 3, 7. CircRNA are generated primarily through a type of alternative RNA splicing called ‘backsplicing’, in which a splice donor splices to an upstream acceptor rather than a downstream acceptor 10. RNA transcripts that are backspliced into loops are not a result of splicing errors; convincing evidence have demonstrated the biogenesis of circRNA is actively regulated by RNA‐binding proteins and specific or repetitive sequences. The biological functions and molecular roles have been characterized for several circRNAs 11, 12, 13; however, the expression profile of circRNA is still elusive for many kinds of diseases.

The advantages of high‐throughput RNA sequencing lead to explore transcriptome in various species. The expression profiles of circRNA and lncRNA have been investigated in numerous biological processes and diseases 14, 15, 16. Ribosome RNA (rRNA) accounts for nearly 70% of mammalian transcriptome, and these rRNA waste a lot of sequencing resources. Poly(A) enrichment is a common approach to avoid rRNA contamination for cDNA library construction during RNA sequencing. Poly(A) enrichment would miss RNA transcripts without poly(A) tail, like circRNA and a part of lncRNA. While ribosomal RNA‐depleted RNA‐seq provides an unbiased selection, regardless of poly(A) tail, and rRNA‐depleted RNA‐seq has been used to investigated lncRNA and circRNA expression 13.

Lung cancer is the first leading cause of cancer‐related death worldwide, and the prognosis is still poor in spite of the advantages achieved in surgery and target therapy 17. Lung adenocarcinoma (LUAD) is the major histological type of lung cancer, especially in China 18. Thus, it is essential to further investigate the molecular basis of LUAD and identify effective diagnostic and prognostic biomarker. In this study, we investigated mRNA, lncRNA, and circRNA expression profiles in LUAD by ribosomal RNA‐depleted RNA sequencing. Analysis of RNA‐seq data revealed that a set of lncRNA and circRNA are differentially expressed in LUAD, circRNA are often of low abundance, and multiple circRNA could be generated from one host gene.

## Results

### Overview of RNA‐seq datai

**Figure 1:**
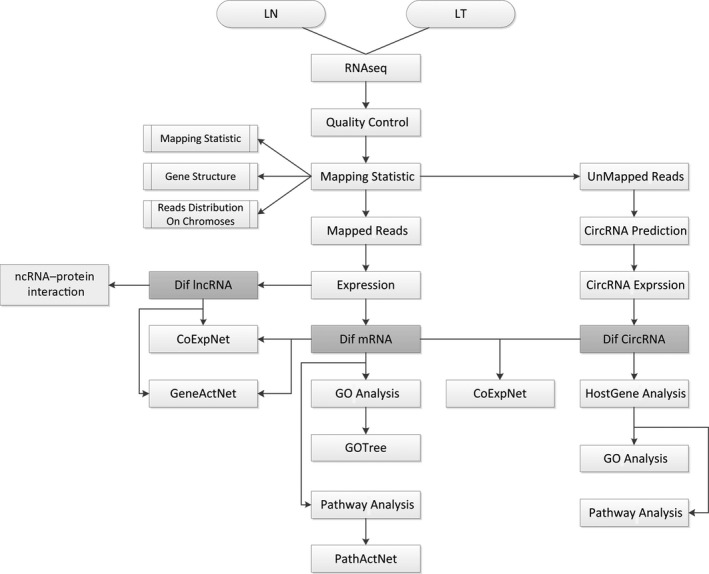
The overall data analysis workflow. LN: normal lung tissues; LT: lung tumor tissues; Dif: differentially expressed; GeneActNet: gene action network; PathActNet: pathway action network.

**Figure 2:**
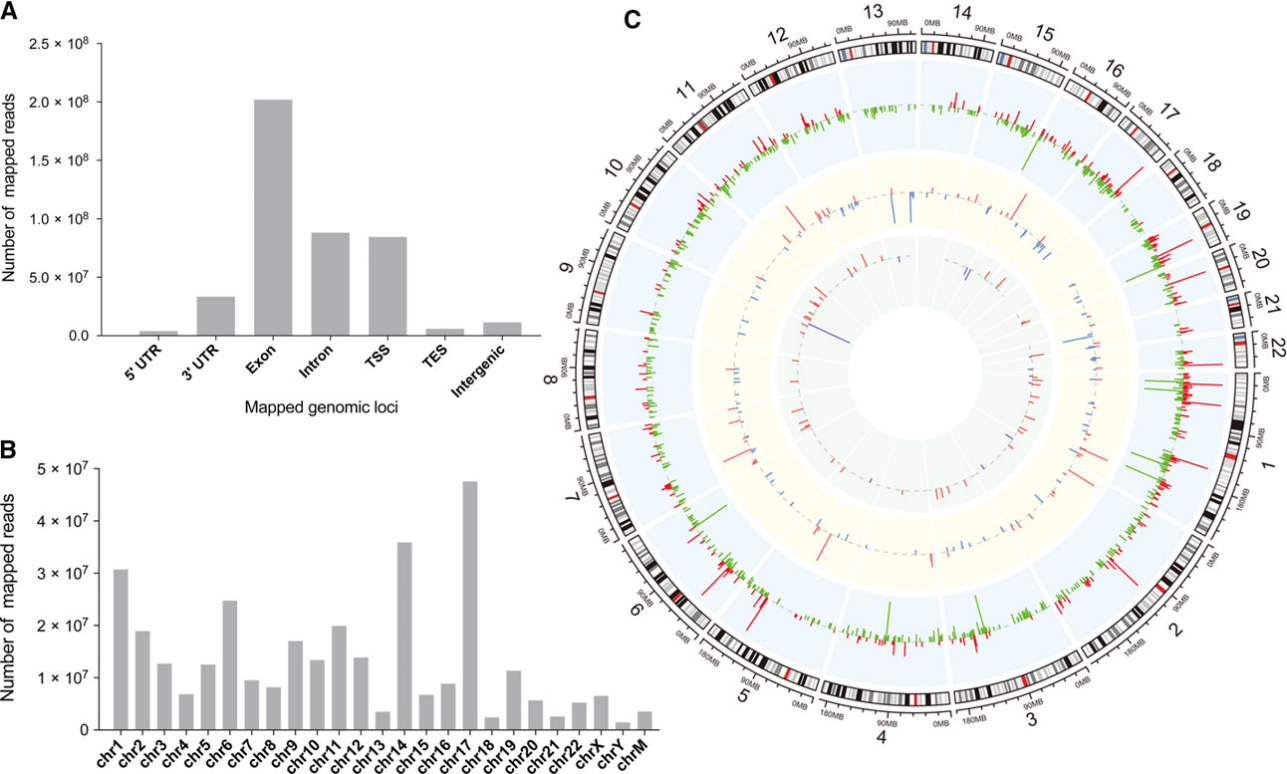
Overview of RNA‐seq data. The majority of reads were mapped to exon, intron, and TSS, while 5′UTR and TES had least mapped reads (A). The number of reads mapped to each chromosome (B). Overview of differentially expressed circRNA, lncRNA, and mRNA and their chromosomal location (C); blue and green: downregulation; red: upregulation; inner circle: circRNA; middle circle: lncRNA; outer circle: mRNA.

The overall data analysis workflow is shown in Fig. 1. Total RNA of LUAD and lung nontumor tissues were extracted and then subjected to rRNA depletion. Reads were first mapped to genome to analyze expression of mRNA and lncRNA, after that the unmapped reads were subjected to circRNA prediction algorithm to identify circRNA expression profile, then the interaction between lncRNA, circRNA, and mRNA were calculated; last, function enrichment analyses were performed to predict potential biological functions of the differentially expressed genes. Most reads were mapped to exon, intron, and TSS, while reads mapped to 5′UTR had lowest abundance (Fig. 2A). Chr18, chr14, and chr1 are the three chromosomes that mapped with most reads, and notably, a number of reads from mitochondria were detected (Fig. 2B). Figure 2C summarizes the differentially expressed circRNA, lncRNA, and mRNA between LUAD and nontumor tissues.

### Expression profiles of coding genes

**Figure 3:**
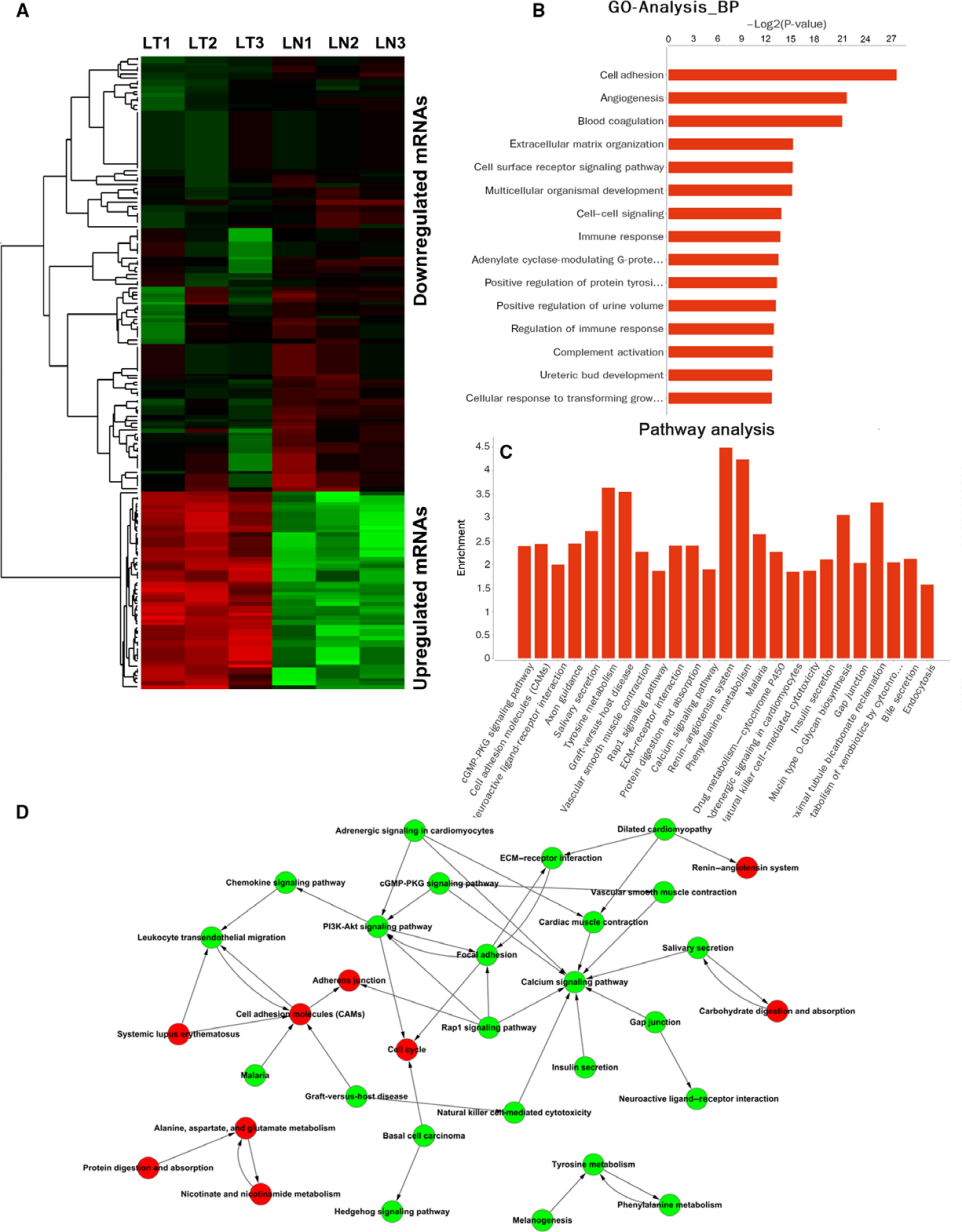
Differentially expressed mRNA in LUAD. The heatmap of top 200 differentially expressed mRNA in LUAD (A); read: upregulation in LUAD, green: downregulation in LUAD. GO analysis of differentially expressed mRNA (B), BP: biological processes. KEGG pathway analyses of differentially expressed mRNA (C). The relationship between enriched pathways was presented (D). Calcium signaling pathway, cell adhesion molecules, PI3K‐Akt signaling pathway, and focal adhesion were the core pathways enriched. Red: upregulated pathways in LUAD; green: downregulated pathways in LUAD.

A number of 20 382 mRNAs were detected, and 1282 were differentially expressed between LUAD and adjacent nontumor tissues (Fig. 3A). Gene ontology (GO) analyses suggested the differentially expressed genes were associated with molecular functions of calcium ion biding, receptor activity, transmembrane signaling receptor activity, and other important functions (Fig. S1A). GO cellular component analyses indicated products of these genes were mostly located at extracellular matrix and plasma membrane (Fig. S1A). GO biological processes showed cell adhesion, angiogenesis, extracellular matrix organization, and cell surface receptor signaling pathways were most enriched among the differentially expressed mRNA (Fig. 3B). The abovementioned processes were all closely associated with caner metastasis. Further investigation of these processes showed that cell–cell adhesion and angiogenesis were the core processes of GO tree (Fig. S1B). Kyoto encyclopedia of genes and genomes (KEGG) pathway analyses suggested that cGMP‐PKG signaling pathway, cell adhesion molecules (CAMs), neuroactive ligand–receptor interaction were most enriched among the differentially expressed genes (Fig. 3C). Calcium signaling pathway, cell adhesion molecules, PI3K‐Akt signaling pathway, and focal adhesion were the core pathways enriched (Fig. 3D); and these four pathways were also related with cancer metastasis.

### LncRNA expression in LUAD

**Figure 4:**
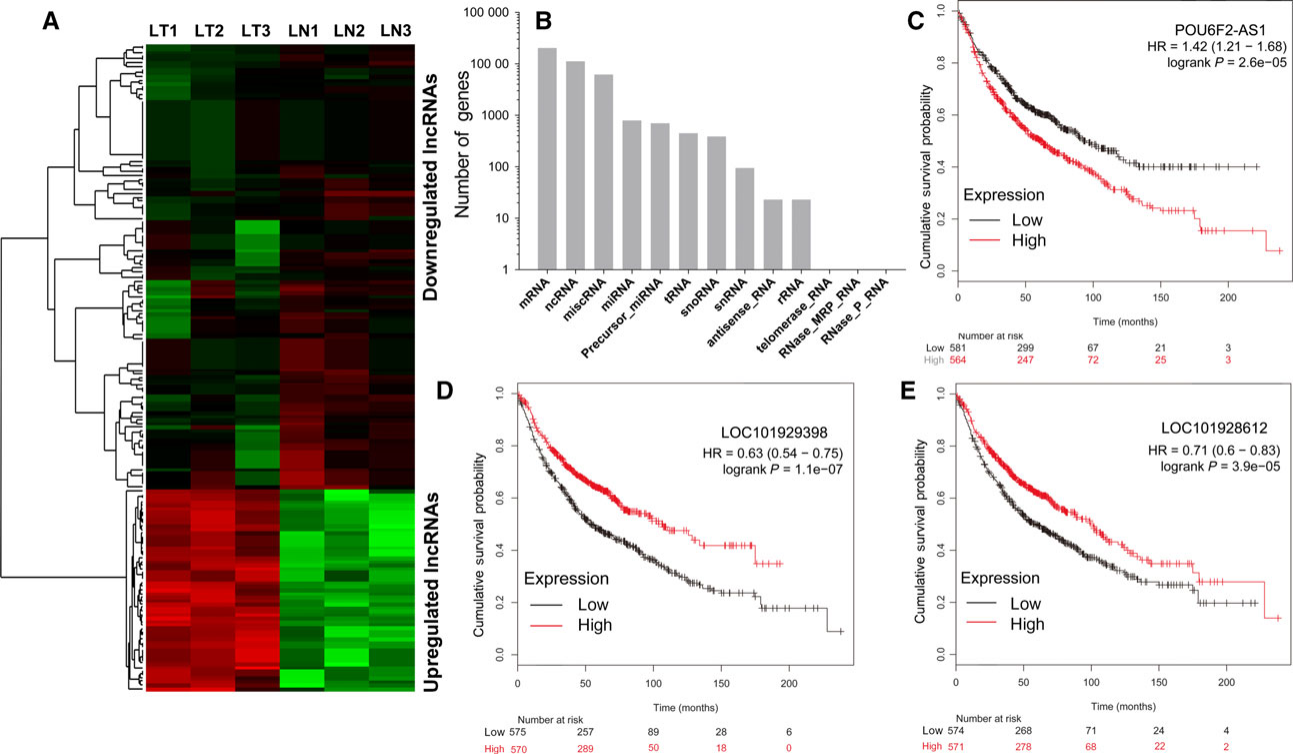
Differentially expressed lncRNA in LUAD. Heatmap of differentially expressed lncRNA; 244 lncRNA were differentially expressed in LUAD according to FC > 2 or < 0.5 and FDR < 0.05 (A). Transcript type of all mapped reads (B). The expression of lncRNA POU6F2‐AS1 (C), LOC101929398 (D), and LOC101928612 (E) is correlated with survival of lung cancer patients.

LncRNA expression profiles were also characterized and the expression of 19 023 lncRNA was analyzed, of which 244 lncRNA were differentially expressed in LUAD (Fig. 4A; Table S3). The transcript types of all mapped reads are shown in Fig. 4B, which showed ncRNA accounted for about one‐third of all mapped reads. AFAP1‐AS1, BLACAT1, and LOC101928245 were the three top upregulated lncRNA in LUAD; FENDRR, LOC400406, and LINC00842 were the three most downregulated lncRNA in LUAD compared with paired nontumor tissues. Our team and other researchers have reported that AFAP1‐AS1 19 and BLACAT1 16 were significantly upregulated and play important roles in lung cancer. In addition, the decreased expression of FENDRR in lung cancer has also been reported 20. The aberrantly expressed lncRNA are usually associated with clinical characteristics and survival of lung cancer patients, and the high expression of AFAP1‐AS1 and BALCAT1 is correlated with poor survival of lung cancer patients. By an online tool, we found these differentially expressed lncRNA were associated with survival of lung cancer patients, such as POU6F2‐AS1, LOC101929398, and LOC101928612 (Fig. 4C–E). These findings suggested these lncRNA might play important roles in lung cancer.

LncRNA–protein interaction was predicted, and the results suggested several lncRNA might interact with protein ELAVL1 (Fig. S2A). LncRNA–mRNA co‐expression network (CoExpNet) was constructed to predict potential biological functions of lncRNA. As shown, the structure of CoExpNets was different in LUAD and nontumor tissues. As shown, lncRNA BALCAT1, LOC101929398, and MYO16‐AS1 were key nodes and have highest degrees in the network. Considering the significant differential expression of BALCAT1 and LOC101929398 and their association with lung cancer prognosis, it is highly possible that lncRNA with high degrees in the CoExpNet might have vital functions in the pathological process of LUAD.

### circRNA expression in LUAD

The circRNA prediction algorithm identified 9340 distinct circRNA candidates (≥ 2 backspliced reads). Compared with online databases, there are 5750 circRNA overlapped with current database and 3590 novel circRNA were identified from our RNA‐seq data. The identified novel circRNAs are provided in Appendix S1.

**Figure 5:**
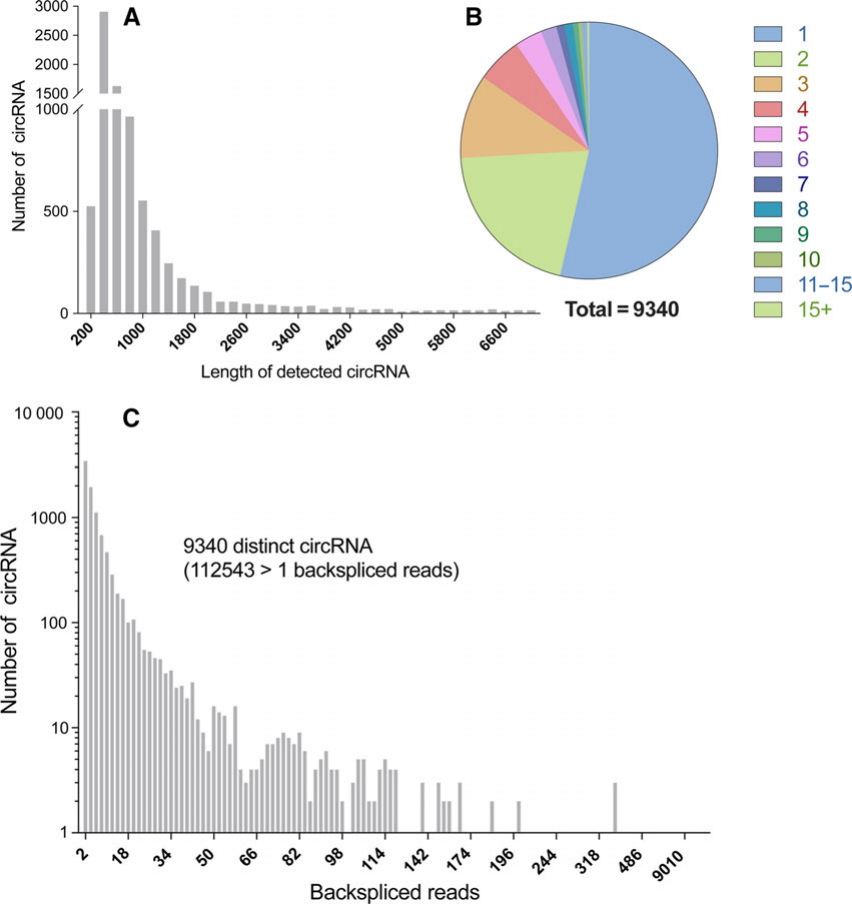
Overview of circRNA characteristics. The median length of detected circRNA was ~ 530 nt (A). The distribution of circRNA according to the number of circRNA derived from one host gene. Multiple circRNA could be generated from one host gene, while more than half of circRNA were derived from on unique gene (B). CircRNA are in general of low abundance, and most circRNA had < 10 detected backspliced reads (C).

The length of most circRNA were < 1000 nt (6579 circRNA are smaller than 1000 nt) and the median length was ~ 530 nt (Fig. 5A), which was consistent with previous report 13, 21. The abundance of circRNA was lower than mRNA and lncRNA. One hundred twelve thousand five hundred forty‐three backspliced reads were detected, and more than half circRNA had < 10 detected reads (Fig. 5B). Multiple circRNA could be produced from one host gene (Fig. 5C), and MACF1 could generate 26 distinct circRNA. In addition, many genes only generate one circRNA; about one‐fourth of detected circRNA were the only circular product of their host genes (2307 of 9340 circRNA, Fig. 5C).

**Figure 6:**
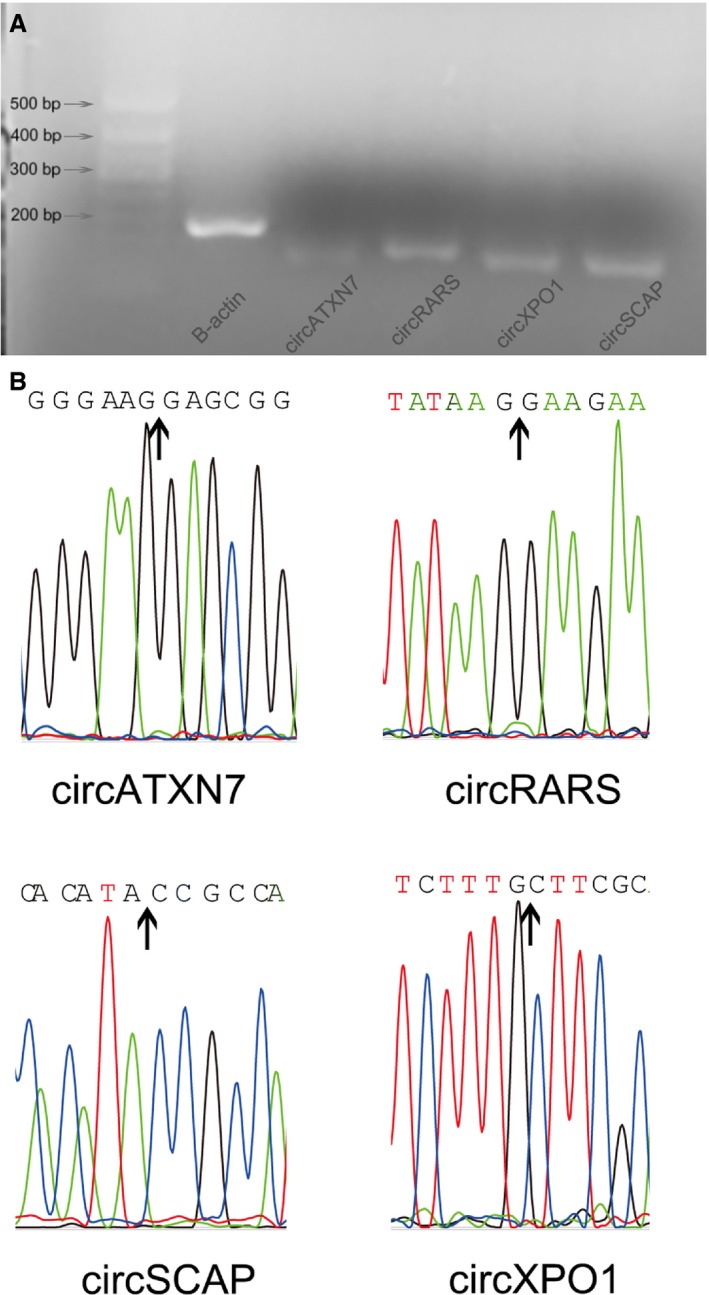
Validation of backsplicing of circRNA. Each pair of primers amplified a single product. B‐actin served as positive control (A). Sanger sequencing confirmed that the backsplicing junction site was detected for each circRNA.

To confirm the backspliced reads were true circular, not trans‐splicing products, divergent primers were designed for four frequently differentially expressed circRNA candidates. Each primer pair amplified a single distinct product of expected size from A549 cell cDNA (Fig. 6A), and Sanger sequencing confirmed the splicing junction site (Fig. 6B).

With the threshold (a) fold change (FC) > 2 or < 0.5, and (b) false discovery rate (FDR) < 0.05, 56 differentially expressed circRNA were filtered (Table S4). Co‐expression between circRNA and mRNA were calculated to predict potential biological functions of these circRNA, as it is suggested genes have same biological functions or within the same pathways may have similar expression pattern. As shown, several well‐known oncogenes were co‐expressed with circRNA, such as Wnt3A, CDK1, and BUB1, indicating these circRNA might participate in the pathological process of lung cancer (Fig. S3).

## Discussion

Although found decades ago, noncoding RNA have not been research focal points till recent years. LncRNA expression profile in lung cancer has been widely reported 16, but the expression pattern of circRNA is still elusive in lung cancer. In the current study, we comprehensively analyzed mRNA, lncRNA, and circRNA expression in LUAD by rRNA‐depleted RNA‐seq. We found 244 lncRNA were differentially expressed in LUAD and identified 3590 novel circRNA, and 56 differentially expressed circRNA.

For the biogenesis of circRNA, several mechanisms have been proposed. Bachmayr *et al*. 14 observed a negative correlation of global circRNA abundance and proliferation and hypothesize that circRNA could accumulate in nonproliferating cells. By investigating circRNA during human epithelial–mesenchymal transition, Conn 22 found many abundant circRNA were dynamically regulated by the splicing factor, Quaking, suggesting the biogenesis of circRNA could be regulated by RNA‐binding proteins. For exonic circRNA, the circularization may be facilitated by complementary sequences, such as Alu elements, in the flanking introns, and by proteins modulating the proper interaction between upstream and downstream introns 11, 23.

In our study, we observed the majority of circRNA were derived from exons and introns via circularization of pre‐mRNA, which is consistent with previous reports that most circRNA are derived from coding sequences 13. Analysis of circRNA number and their host genes revealed that multiple circRNA could be generated from one host gene. We observed MACF1 could generate 26 distinct circRNA, while previous reports showed PTK2 could generate 47 distinct circRNA. The length of most circRNA was around 200–800 nt, consistent with previous reports that the median length of circRNA was around 500 nt 13, 21.

Mounting evidence has proved that circRNA play important roles in various biological processes and diseases 5, 9, and circRNA function as a ‘microRNA sponge’ in most cases 24. A striking example is CDR1as 25, which is highly abundant in brain and harbors more than 60 conserved binding sites of miR‐7. To predict the potential function of abundantly expressed circRNA, we constructed CoExpNet for circRNA and mRNA. As shown, many oncogenes were co‐expressed with circRNA, such as Wnt3a, and CDK1. It is possible to infer these circRNA might be involved in these biological processes.

Molecular studies of eukaryotic RNA usually begin with poly(A) selection, which miss a set of lncRNA without poly(A) tail. In the current study, we analyzed lncRNA expression profile in LUAD by rRNA‐depleted RNA‐seq, which may help the detection of lncRNA without poly(A) tail. To our knowledge, this is the first study employed ribosomal RNA‐depleted RNA‐seq in LUAD. AFAP1‐AS1 19, BLACAT1 16, 26, and FENDRR 20 were the top differentially expressed lncRNA in LUAD, which was consistent with our previous work and reports of other researchers, indicating the RNA‐seq data were reliable. LncRNA–mRNA CoExpNet was constructed to predict biological functions of lncRNA. In the CoExpNet of LUAD tissues, BALCAT1 and LOC101929398 were of highest degrees and located at the centers of network. Accordingly, BALCAT1 has been demonstrated as an oncogenic gene in lung cancer; Kaplan–Meier curve suggested high expression of LOC101929398 was a poor prognostic factor for lung cancer. These lines of evidence indicated lncRNA LOC101929398 might be a proto‐oncogenic gene in LUAD. With the help of co‐expression, we might identify lncRNA that would play important roles in LUAD.

## Conclusion

We analyzed mRNA, lncRNA, and circRNA expression in LUAD by rRNA‐depleted RNA‐seq. AFAP1‐AS1, BLUADAT1, and LOC101929398 were top upregulated lncRNA in LUAD. We detected the expression of 9340 circRNA, including 3570 novel circRNA, demonstrating that circRNA are widely expressed in LUAD.

## Materials and methods

### Patients and tissue samples

This study was approved by the Ethics Committee of Cancer Institute of Jiangsu Province. Paired LUAD tissues and adjacent nontumor tissues were obtained from patients who received surgical resection of NSCLC between 2012 and 2014 at the Department of Thoracic Surgery of Cancer Institute of Jiangsu Province, China. The pathology classification of paired tumor and nontumor tissues was confirmed by experienced pathologists. Clinical and pathological characteristics were also collected for each patient. Informed written consent was obtained from all patients included in this study. Nine pairs of LUAD and nontumor tissues were collected, and the RNA of every three LUAD or nontumor tissues were pooled for RNA sequencing; six samples were sequenced overall (three samples of LUAD and three samples of nontumor tissues). Clinical characteristics of LUAD patients and RNA‐seq sample are provided in Table S1. RNA‐seq data are being uploaded to Gene Expression Omnibus database and will be published once finished uploading.

### Cell culture and PCR

A549 cells were cultured in RPMI 1640 medium (GIBCO, Invitrogen, Shanghai, China) supplemented with 10% FBS (GIBCO‐BRL, Invitrogen, Carlsbad, CA, USA), 100 U·mL^−1^ penicillin, and 100 mg·mL^−1^ streptomycin (KeyGEN) in humidified air at 37 °C with 5% CO_2_. The total RNA was extracted from A549 cells with TRIzol reagent (Invitrogen, Grand Island, NY, USA), according to the manufacturer's protocol; 1 mg total RNA was reverse‐transcribed in a final volume of 20 μL using PrimerScript RT Master Mix (Takara, Dalian, China). qRT‐PCR was performed as previously described. Primers used are provided in Table S2. The PCR products were subjected to agarose electrophoresis and Sanger sequencing.

### Sample preparation

Total RNA was extracted from LUAD and paired nontumor tissues by TRIzol reagent (Invitrogen, Shanghai, China) separately. The RNA quality was checked by Bioanalyzer 2200 (Agilent, Santa Clara, CA, USA) and kept at −80 °C. The RNA with RIN > 8.0 are right for rRNA depletion. rRNA was depleted by GeneRead rRNA Depletion Kit (QIAGEN, Venlo, Germany).

### Library construction and RNA sequencing:

The cDNA libraries for single‐end sequencing were prepared using Ion Total RNA‐Seq Kit v2.0 (Thermo Fisher Scientific, Inc., Waltham, MA, USA) according to the manufacturer's instructions. The cDNA libraries were then processed for the proton sequencing process according to the commercially available protocols. Samples were diluted and mixed, and the mixture was processed on a OneTouch 2 instrument (Thermo Fisher Scientific) and enriched on a OneTouch 2 ES station (Thermo Fisher Scientific) for preparing the template‐positive Ion PI™ Ion Sphere™ Particles (Thermo Fisher Scientific) according to Ion PI™ Template OT2 200 Kit v2.0 (Thermo Fisher Scientific). After enrichment, the mixed template‐positive Ion PI™ Ion Sphere™ Particles of samples were loaded onto 1 P1v2 Proton Chip (Thermo Fisher Scientific) and sequenced on proton sequencers according to Ion PI Sequencing 200 Kit v2.0 (Thermo Fisher Scientific) by NovelBio Corp. Laboratory, Shanghai, China.

### RNA sequencing data mapping

Mapping of single‐end reads. Before read mapping, clean reads were obtained from the raw reads by removing the adaptor sequences, reads with > 5% ambiguous bases (noted as N), and low‐quality reads containing more than 20 percent of bases with qualities of < 13. The clean reads were then aligned to human genome (version: GRCh37) using the mapsplice program (v2.1.8). In alignment, preliminary experiments were performed to optimize the alignment parameters (–s 22 –p 12 –ins 6 –del 6 –noncanonical) to provide the largest information on the alternative splicing events 27.

### CircRNA identification and quantification

The pipeline ‘acfs’, which was publicly available at https://code.google.com/p/acfs/, was used to identified circRNA in each samples including following steps 8:

- Unmapped reads collection: bowtie2 version 2.2.5 28 was used as the mapping method to the respective reference genome [GRCH37.p13 NCBI].
- CircRNA identification: Unmapped reads were collected to identify the circRNA utilizing BWA mem (bwa mem –t 1 –k 16 –T 20): Partial alignments of segments within a single read that mapped to (a) regions on the same chromosome and no more than 1 Mb away from each other (b) on the same strand (c) but in reverse order were retained as candidates supporting head‐to‐tail junction. The strength of potential splicing sites supported by these candidate head‐to‐tail junction reads was then estimated using MaxEntScan33. The exact junction site was determined by selecting the donor and acceptor sites with the highest splicing strength score. Candidate circRNA were reported if the head‐to‐tail junction was supported by at least two reads and the splicing score was ≥ 10.
- Expression analysis: To estimate the expression of circRNA, we realigned all the unmapped reads to the circRNA candidates using the BWA mem under following parameter (bwa mem –t 1 –k 16 –T 20). As for most of the circRNA there is no direct evidence for their exact sequence, we filled in the sequence using existing exon annotation. Sequence at the 5′ end was concatenated to the 3′ end to form circular junctions. Reads that mapped to the junction (with an overhang of at least 6 nt) were counted for each candidate.

### Differentially expressed genes

We applied DESeq algorithm 29 to filter the differentially expressed genes. FC and FDR were used to filter differentially expressed genes under the following criteria 30: (a) FC > 2 or < 0.5; (b) FDR< 0.05.

### GO analysis

Gene ontology analysis was performed to facilitate elucidating the biological implications of differentially expressed mRNA 31. We downloaded the GO annotations from NCBI (http://www.ncbi.nlm.nih.gov/), UniProt (http://www.uniprot.org/), and the GO (http://www.geneontology.org/). Genes annotated by integrated GO database were set up as the background genes and based on the background information; Fisher's exact test was applied for the GO analysis with significant *P*‐value calculated, and FDR was used to correct the *P*‐values.

### Pathway analysis

Pathway analysis was used to find out the significant pathway of the differential genes according to KEGG database. We turn to the Fisher's exact test to select the significant pathway, and the threshold of significance was defined by FDR < 0.05 32.

### GO tree

The GO is structured as a directed acyclic graph, and each term has defined relationships to one or more other terms. GO tree is built based on the GO directed acyclic graph to provide user‐friendly data navigation and visualization. We selected the significant GO term (*P*‐value < 0.01) in GO analysis based on the up and down differentially expressed genes to construct the GO tree to summarize the function affected in the experiment 33.

### Pathway network

Kyoto encyclopedia of genes and genomes 34 included metabolism, membrane transport, signal transduction, and cell cycle pathways. We picked the genes in enriched biological pathway and using cytoscape (version 3.2.1) for graphical representations of pathways 35.

### Co‐expression network

We present gene CoExpNets to find the interactions among genes 36. Gene CoExpNets were built according to the normalized signal intensity of specific expression genes. For each pair of genes, we calculate the Pearson correlation and choose the significant correlation pairs to construct the network.

Within the network analysis, degree centrality is the most simplest and important measures of the centrality of a gene within a network that determine the relative importance. Degree centrality is defined as the link numbers one node has to the other 37. Moreover, to investigate some properties of the networks, W‐cores in graph theory were introduced as a method of simplifying graph topology analysis. A W‐core of a network is a subnetwork in which all nodes are connected to at least W other genes in the subnetwork. A W‐core of a protein–protein interaction network usually contains cohesive groups of proteins 38.

The purpose of network structure analysis is to locate core regulatory factors. In one network, core regulatory factors connect most adjacent genes and have biggest degrees. While considering different networks, core regulatory factors were determined by the degree differences between two class samples 39. They always own the biggest degree differences.

### LncRNA–protein interaction

Use NPInter (http://www.bioinfo.org/NPInter) to build the network of lncRNA and proteins according to functional interactions between noncoding RNA (except tRNA and rRNA) and biomolecules (proteins, RNA and DNA), which are experimentally verified. Interactions are manually collected from publication in peer‐reviewed journals, followed by an annotation process against known databases including NONCODE (http://www.noncode.org/), miRBase (http://www.mirbase.org/), and UniProt (http://www.uniprot.org/). NPInter introduces a classification of the functional interaction data, which is based on the functional interaction process the ncRNA takes part in. NPInter also provides an efficient search option, allowing discovery of interactions, related publications, and other information 40.

## Author contributions

MQ, RY, LX, and JW designed experiments. MQ, XD, XL, SZ, QH, CF, SW, SW, ML, and JX performed the experiments. MQ, SW, RY, LX, ML, FY, and JW performed the data analysis. MQ, XD, XL, and SZ wrote the manuscript.

## Supporting information


**Fig. S1**. GO analysis of differentially expressed mRNA.

**Fig. S2**. LncRNA‐protein interaction network.

**Fig. S3**. CoExpNet between differentially expressed circRNA and mRNA.

**Table S1**. Clinical characteristics of LUAD patients and RNA‐seq sample ID.

**Table S2**. Primers used in this study.

**Table S3**. Differentially expressed lncRNAs in LUAD.

**Table S4**. Differentially expressed circRNAs in LUAD.

**Appendix S1.** Sequences of detected novel circRNA candidates.
