# Supplementary figures and images for: Profiling expression of coding genes, long noncoding RNA, and circular RNA in lung adenocarcinoma by ribosomal RNA‐depleted RNA sequencing
Source: FEBS Open Bio. 2018 Feb 21;8(4):544–55. doi: 10.1002/2211-5463.12397 (PMC5881538; doi:10.1002/2211-5463.12397)

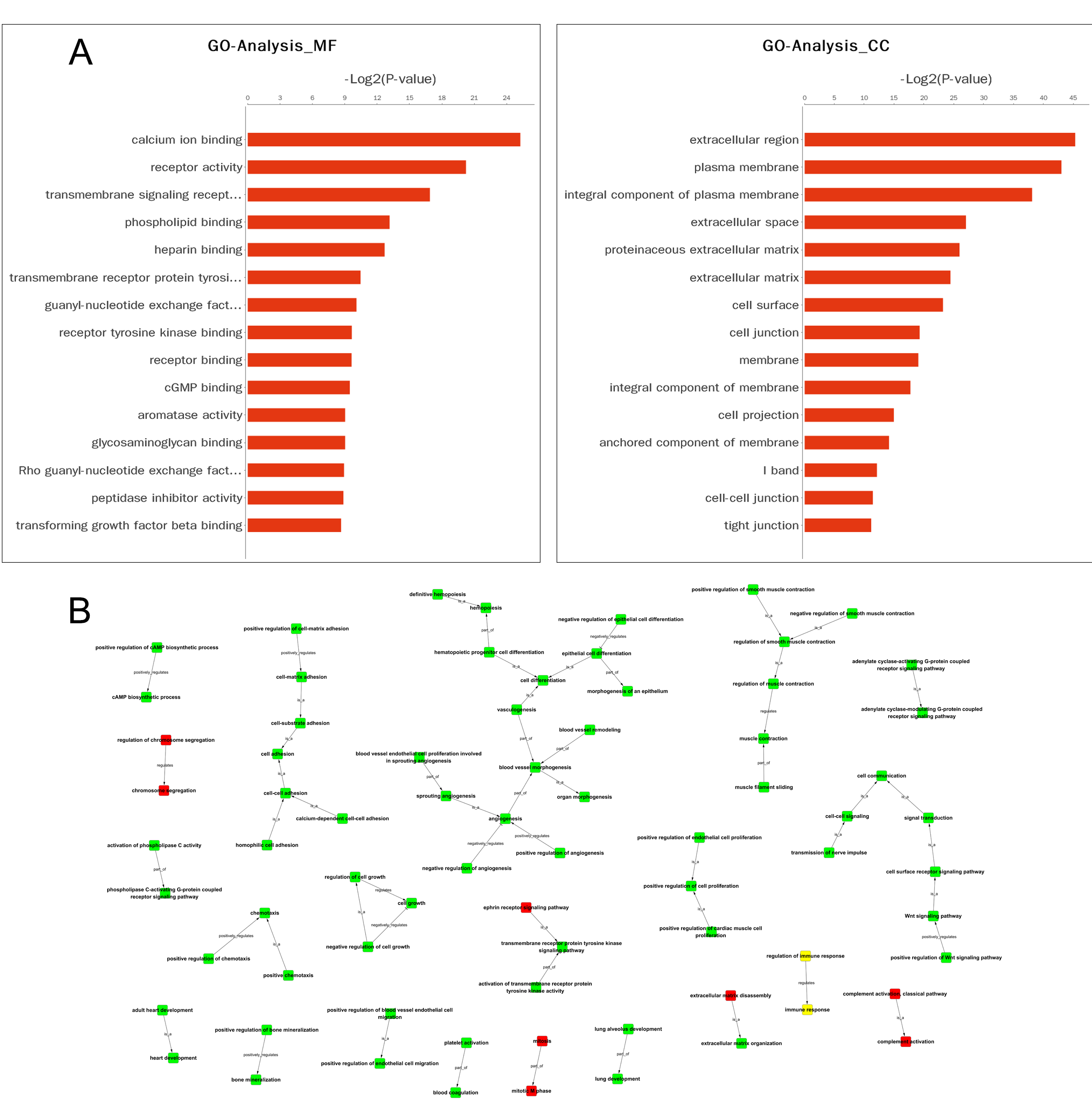

Supplement: Supplementary file 1 — Fig. S1. GO analysis of differentially expressed mRNA. [file FEB4-8-544-s001.tif]

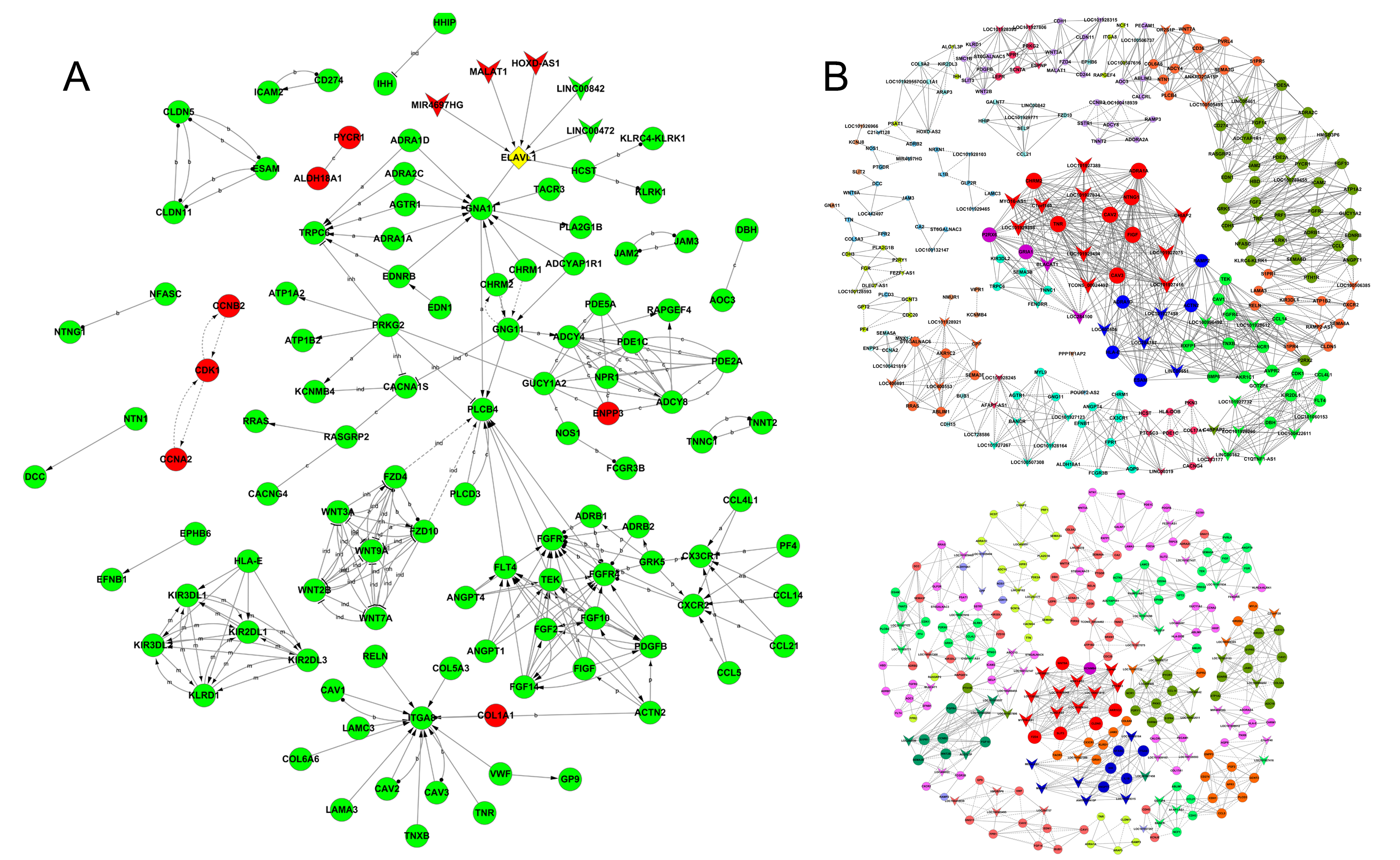

Supplement: Supplementary file 2 — Fig. S2. LncRNA‐protein interaction network. [file FEB4-8-544-s002.tif]
