# Supplementary material for: Profiling expression of coding genes, long noncoding RNA, and circular RNA in lung adenocarcinoma by ribosomal RNA‐depleted RNA sequencing
Source: FEBS Open Bio. 2018 Feb 21;8(4):544–55. doi: 10.1002/2211-5463.12397 (PMC5881538; doi:10.1002/2211-5463.12397)
Supplement: Supplementary file 9 [file FEB4-8-544-s009.docx]

**Appendix S1**. Sequences of detected novel circRNA candidates
