# Supplementary material for: Profiling expression of coding genes, long noncoding RNA, and circular RNA in lung adenocarcinoma by ribosomal RNA‐depleted RNA sequencing
Source: FEBS Open Bio. 2018 Feb 21;8(4):544–55. doi: 10.1002/2211-5463.12397 (PMC5881538; doi:10.1002/2211-5463.12397)
Supplement: Supplementary file 3 — Fig. S3. CoExpNet between differentially expressed circRNA and mRNA. [file FEB4-8-544-s003.pdf]

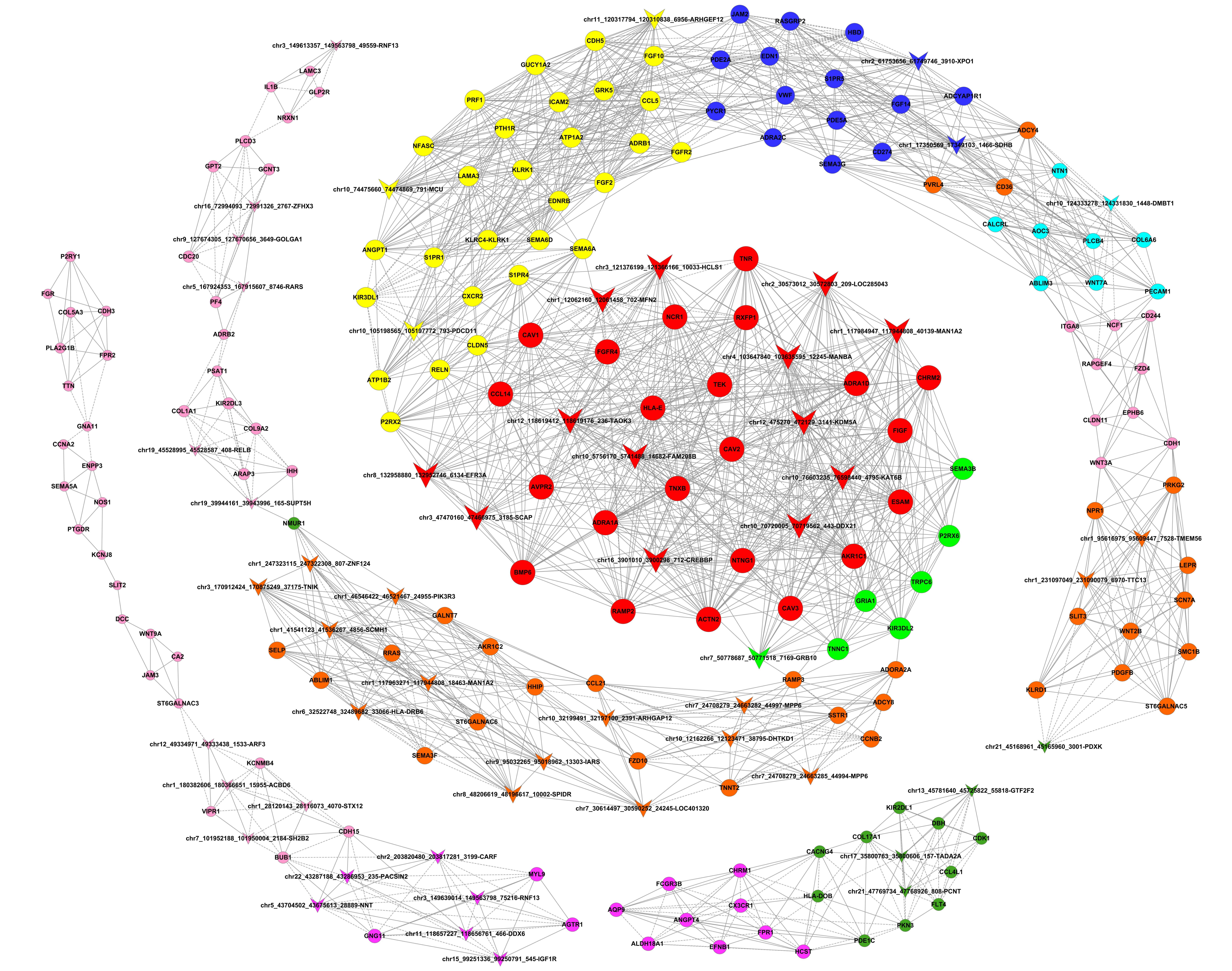

chr11_120317794_120310838_6956-ARHGEF12
JAM2
RASGRP2
HBD
CDH5
FGF10
PDE2A
EDN1
chr2_61753656_61749746_3910-XPO1
GUCY1A2
GRK5
S1PR5
PRF1
ICAM2
CCL5
VWF
PYCR1
FGF14
ADCYAP1R1
PDE5A
PTH1R
ATP1A2
ADRB1
ADRA2C
ADCY4
FGFR2
CD274
chr1_17350569_17349103_1466-SDHB
NFASC
SEMA3G
LAMA3
KLRK1
FGF2
PVRL4
CD36
NTN1
chr10_74475660_74474869_791-MCU
EDNRB
chr10_124333278_124331830_1448-DMBT1
CALCRL
AOC3
PLCB4
COL6A6
ANGPT1
KLRC4-KLRK1
SEMA6D
SEMA6A
S1PR1
TNR
chr3_121376199_121366166_10033-HCLS1
chr2_30573012_30572803_209-LOC285043
S1PR4
ABLIM3
WNT7A
PECAM1
KIR3DL1
CXCR2
chr1_12062160_12061458_702-MFN2
NCR1
RXFP1
CD244
ITGA8
NCF1
chr10_105198565_105197772_793-PDCD11
chr1_117984947_117944808_40139-MAN1A2
CLDN5
CAV1
FGFR4
chr4_103647840_103635595_12245-MANBA
RAPGEF4
FZD4
RELN
ATP1B2
TEK
ADRA1D
CHRM2
CCL14
HLA-E
chr12_118619412_118619176_236-TAOK3
chr12_475270_472129_3141-KDM5A
P2RX2
FIGF
CLDN11
EPHB6
CAV2
chr10_5756170_5741488_14682-FAM208B
CDH1
WNT3A
chr8_132958880_132952746_6134-EFR3A
SEMA3B
chr10_76603235_76598440_4795-KAT6B
AVPR2
TNXB
ESAM
PRKG2
chr3_47470160_47466975_3185-SCAP
NPR1
ADRA1A
chr10_70720005_70719562_443-DDX21
P2RX6
chr1_95616975_95609447_7528-TMEM56
chr16_3901010_3900298_712-CREBBP
NTNG1
AKR1C1
LEPR
BMP6
TRPC6
chr1_231097049_231090079_6970-TTC13
RAMP2
ACTN2
CAV3
GRIA1
SCN7A
SLIT3
KIR3DL2
WNT2B
TNNC1
SMC1B
chr7_50778687_50771518_7169-GRB10
PDGFB
KLRD1
ST6GALNAC5
chr21_45168961_45165960_3001-PDXK
chr3_149613357_149563798_49559-RNF13
LAMC3
IL1B
GLP2R
NRXN1
PLCD3
GPT2
GCNT3
chr16_72994093_72991326_2767-ZFHX3
chr9_127674305_127670656_3649-GOLGA1
CDC20
chr5_167924353_167915607_8746-RARS
PF4
ADRB2
PSAT1
KIR2DL3
COL1A1
COL9A2
chr19_45528995_45528587_408-RELB
ARAP3
IHH
chr19_39944161_39943996_165-SUPT5H
NMUR1
P2RY1
FGR
COL5A3
CDH3
PLA2G1B
FPR2
TTN
GNA11
CCNA2
ENPP3
SEMA5A
NOS1
PTGDR
KCNJ8
SLIT2
DCC
WNT9A
CA2
JAM3
ST6GALNAC3
chr1_247323115_247322308_807-ZNF124
chr3_170912424_170875249_37175-TNIK
chr1_46546422_46521467_24955-PIK3R3
GALNT7
chr1_41541123_41536267_4856-SCMH1
SELP
RRAS
AKR1C2
ABLIM1
chr1_117963271_117944808_18463-MAN1A2
HHIP
CCL21
RAMP3
ADORA2A
chr6_32522748_32489682_33066-HLA-DRB6
ST6GALNAC6
chr7_24708279_24663282_44997-MPP6
ADCY8
chr10_32199491_32197100_2391-ARHGAP12
SSTR1
chr10_12162266_12123471_38795-DHTKD1
CCNB2
SEMA3F
chr9_95032265_95018962_13303-IARS
FZD10
chr7_24708279_24663285_44994-MPP6
TNNT2
chr8_48206619_48196617_10002-SPIDR
chr7_30614497_30590252_24245-LOC401320
chr12_49334971_49333438_1533-ARF3
KCNMB4
chr1_180382606_180366651_15955-ACBD6
chr1_28120143_28116073_4070-STX12
VIPR1
CDH15
chr7_101952188_101950004_2184-SH2B2
BUB1
chr2_203820480_203817281_3199-CARF
chr22_43287188_43286953_235-PACSIN2
MYL9
chr3_149639014_149563798_75216-RNF13
chr5_43704502_43675613_28889-NNT
AGTR1
GNG11
chr11_118657227_118656761_466-DDX6
chr15_99251336_99250791_545-IGF1R
chr13_45781640_45725822_55818-GTF2F2
KIR2DL1
DBH
COL17A1
CDK1
CACNG4
chr17_35800763_35800606_157-TADA2A
CHRM1
CCL4L1
FCGR3B
chr21_47769734_47768926_808-PCNT
HLA-DOB
CX3CR1
FLT4
AQP9
ANGPT4
FPR1
PKN3
PDE1C
ALDH18A1
EFNB1
HCST
